# Copper Recovery from Metallurgical Slags Using Biomass in the Form of Fruit Seeds/Stones as a Reducing Agent

**DOI:** 10.3390/molecules31193550

**Published:** 2026-10-05

**Authors:** Jerzy Łabaj, Agnieszka Fornalczyk, Robert Findorak, Adrian Smagór, Tomasz Matuła, Bartosz Chmiela, Leszek Blacha

**Affiliations:** 1 Silesian University of Technology, 44-100 Gliwice, Poland; agnieszka.fornalczyk@polsl.pl (A.F.); adrian.smagor@polsl.pl (A.S.); tomasz.matula@polsl.pl (T.M.); bartosz.chmiela@polsl.pl (B.C.); leszek.blacha@polsl.pl (L.B.); 2 Technical University of Kosice, 042 00 Sever, Slovakia; robert.findorak@tuke.sk

**Keywords:** recycling of metals, copper metallurgy, biomass, reduction process, efficient use of biomass-derived raw materials

## Abstract

Civilizational development, which depends on the availability of raw materials, has accelerated tremendously in recent decades. This is due to globalization and the growth of emerging markets. It represents a huge opportunity for an innovative approach to both production and consumption. Along with civilizational growth, we also face negative consequences that lead to climate change. To meet the demands regarding climate neutrality, people have started analyzing the possibility of running production processes using substrates that either eliminate or significantly reduce the use of non-renewable resources. Considering the specifics of metal extraction and production, it is important to note that chemistry and thermodynamics play a key role here. This involves, on the one hand, understanding how carbon or its oxides are absorbed, which is essential for plant growth, and on the other, using components of so-called biomass for controlled chemical reactions. These regulations regarding climate neutrality and the type of raw materials are reflected in detail in the relevant European Union directives. Copper production in Poland is a strategic sector of the economy. Considering the scale of production and the consumption of natural resources, developing the possibility of partially replacing them will constitute a process innovation. Research into copper recovery from slag to date has focused primarily on improving the efficiency of the slag reduction process by intensifying bath mixing and improving the mass transfer process in the liquid phase. Given that biomass contains a significant proportion of volatile matter, this may additionally have a beneficial impact on the efficiency of using this type of material in the process. Many parameters still need to be investigated before the obtained results can be tested on a larger scale, enabling transfer to industrial conditions. The study presented the results of research on the potential use of biomass to reduce secondary raw materials coming from the copper industry. A Gibbs free enthalpy analysis was carried out for possible chemical reactions and their likelihood of occurring. As a source of metals, a material similar in composition to slag generated in the copper industry was used. Considering that copper ores contain about 1 wt. %, it makes even more sense to use secondary slag, which contains up to 10 wt. % copper. As part of the work, tests of the pyrometallurgic slag reduction process were carried out with the use of selected types of biomass. The tests were carried out in a metallurgical aggregate, at a temperature of 1300 °C in the time range from 1 to 5 h. The main parameters determining the efficiency of the process were to determine the degree of removal of the main metals, i.e., Cu and Pb, as well as to determine the content of these elements in the secondary slag. Verification of the possibility of replacing coal with biomass in real industrial conditions requires verifying primarily the effectiveness of metal removal as well as the possibility of determining the effect of climate neutrality. The results show the huge potential biomass has in terms of use in metal production processes. Also, in the near future the aspect of climate neutrality in the European Union may become an important factor for the economics of the process and competitiveness in the market.

## 1. Introduction

Biomass is classified as a renewable energy source [1,2]. It consists of all materials of animal and plant origin that can be used to generate energy. It is assumed that energy from biomass accounts for more than 50% of the total energy obtained from renewable energy sources. In the processing of stone fruits, mainly cherries, apricots, peaches, and plums, biomass is produced in the form of pits, the rational management of which is relatively difficult. Due to their hard outer shell, pits decompose very slowly in the natural environment, which hinders their composting process. At the same time, this type of biomass does not pose problems during transport or storage. For years, research has been conducted into the possibility of utilizing pits as an energy feedstock. This includes their direct use as a solid fuel or as a substrate for producing liquid or gaseous biofuels. Another way to utilize pits is to produce activated carbon, edible oils, or cosmetic oils from them, as well as to use them in crushed form as a feed ingredient. Table 1 summarizes, by way of example, the main directions of past research regarding the applications of cherry, apricot, and plum pits.

The available literature data indicating the directions of utilizing various types of biomass in metallurgical processes show that they primarily concern their potential use in metal ore sintering, reduction, and the recovery of metals from secondary raw materials [32]. Biomass can serve as a sustainable alternative to coke, which is commonly used as a fuel in these processes, as well as an alternative reducing agent that lowers CO_2_ emissions. Attempts to utilize biomass in sintering processes have focused on iron ores, as well as manganese and chromite ores, and have mainly involved the use of charcoal or bio-coke [33,34,35,36,37,38]. Biomass has also been used as an additive in the preparation of iron ore pellets [39,40,41]. Research has also been conducted into the possibility of directly injecting biofuel into the blast furnace during the pig iron production process [42,43]. When biomass is injected into the blast furnace nozzle, it simultaneously acts as a fuel and, indirectly, as a reducing agent by participating in the carbon monoxide formation reaction. In recent years, there has also been an expansion of activities in the field of utilizing biomass in metal recycling processes from secondary metal-bearing raw materials, such as slag, dust, and sludge [44,45,46,47,48,49,50,51,52,53,54]. In these processes, biomass is mainly used as a replacement for the carbonaceous materials most commonly used in them, i.e., coke, slack, and anthracite dust. It should also be emphasized that utilizing biomass in these technological processes as a coke substitute allows, on the one hand, for the reduction in pollutant emissions such as SO_2_ and NOx, and on the other hand, it can intensify the reduction process of metal compounds itself through the hydrocarbons released during the process. The latter results from the fact that the main components of biomass, i.e., cellulose, hemicellulose, and lignin, release additional reducing gases, such as hydrogen, methane, and carbon monoxide, during thermal decomposition.

This research article discusses the potential opportunities for utilizing fruit pits in the copper slag recycling process to recover copper and lead. Additionally, the novelty will be the presentation of the possibilities of replacing natural raw materials, such as coke, with biomass, which can be used to recover metals from secondary raw materials and develop climate neutrality guidelines, which would help reduce metal production costs.

## 2. Physicochemical Properties of Fruit Pits

**Figure 1:**
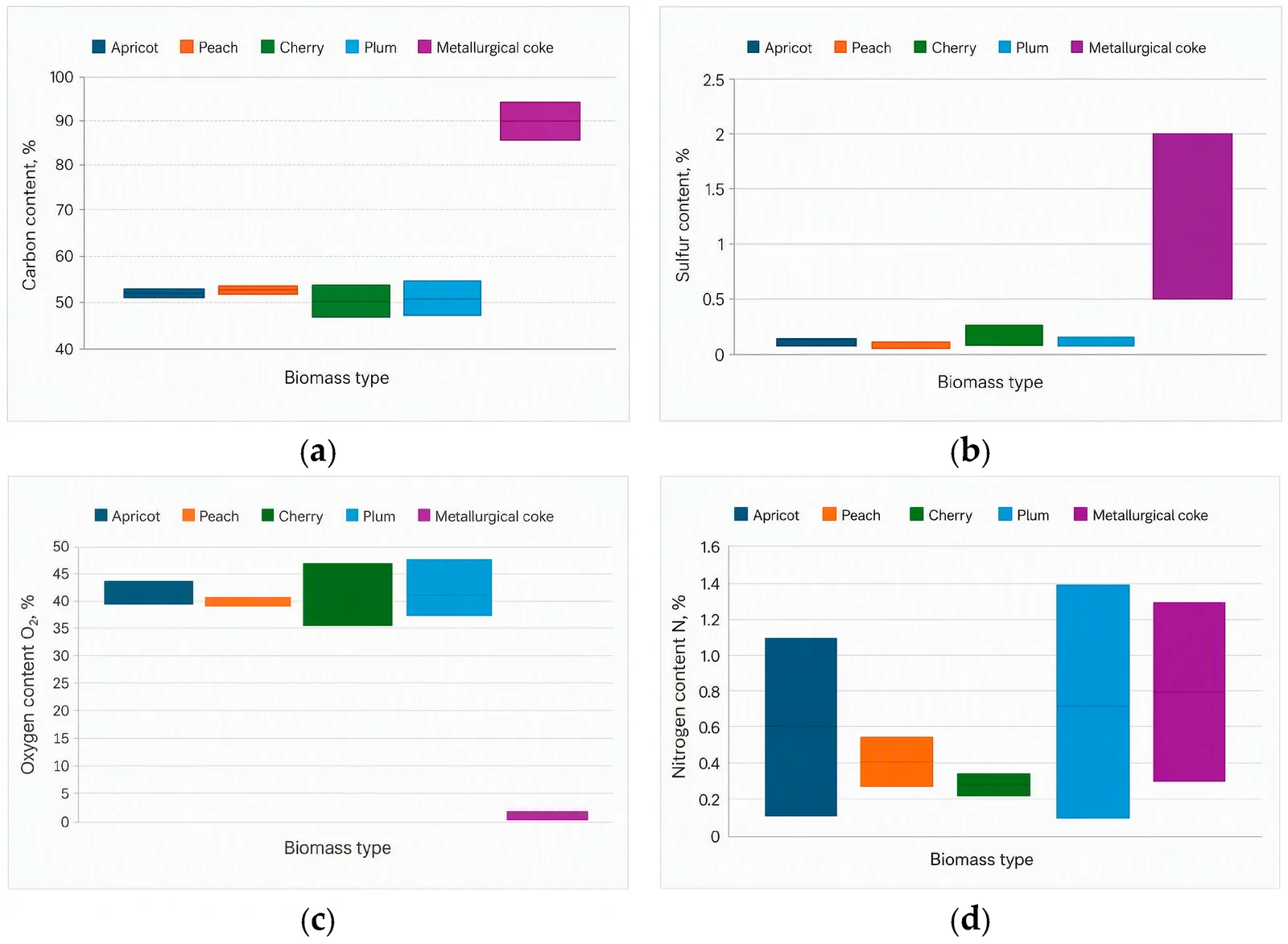
Content of basic components in fruit pits and metallurgical coke: (**a**) carbon, (**b**) sulphur, (**c**) oxygen, (**d**) nitrogen [55,56,57,58,59,60,61,62,63,64].

The primary components of biomass, and therefore also of fruit pits, are carbon, oxygen, and hydrogen. Based on the collected literature data for cherry, apricot, and plum pits, the content ranges for carbon, sulphur, hydrogen, nitrogen, and oxygen were 46.4–54.2 wt.% for carbon; 0.01–0.03 wt.% for sulphur; 5.45–6.63 wt.% for hydrogen; 0.10–1.38 wt.% for nitrogen; and 38.2–48.0 wt.% for oxygen [55,56,57,58,59,60,61,62,63,64]. These data are presented in detail in Figure 1. For comparison purposes, the chemical composition of metallurgical coke was also included in the figures. From analyzing these data, it can be concluded that fruit pits contain nearly half as much carbon as coke and almost four times more oxygen. Consequently, this results in a high volatile matter content in the pits. On the contrary, the sulphur content in fruit pit biomass is significantly lower than in coke, which is environmentally beneficial when it is utilized as a fuel. Furthermore, fruit pits are characterized by a relatively high hydrogen content and a lower nitrogen content compared to metallurgical coke. When analyzing the moisture content in biomass, it should be kept in mind that this is one of the main parameters determining its suitability for use as a fuel. Raw biomass is characterized by a high total moisture content, reaching above 50 wt.%. In the case of the studied fruit pits, the value of this parameter is significantly lower and ranges from 0.18 to 10.7 wt.% [55,56,57,58,59,60,61,62]. Metallurgical coke, on the other hand, is characterized by a moisture content ranging from 2 to 5 wt.%. When analyzing the ash content in fruit pits, it must be remembered that it occurs across a very wide range in biomass. An elevated ash content adversely affects, among other things, the energy value of the fuel and the course of its combustion process. In the case of fruit pits, the ash content is relatively low and ranges from 0.2 to 1.8 wt.%; for coke, the value of this parameter is higher, amounting to approximately 8–15 wt.% [57,65,66,67].

From a practical perspective, introducing biomass into an industrial process, such as copper production, is a new approach that requires determining important process parameters, particularly the introduced components with high volatility. However, the fundamental parameter that can pave the way for industrial application is the recovery efficiency of key elements such as copper and lead. The article presents research in this field.

When analyzing the physicochemical parameters of fruit pits regarding their potential application as fuel and a reducing agent in metal production and recovery processes, attention must also be paid to their heating value. According to the literature data [68], the average value of this parameter for all analyzed types of fruit pits was at a similar level, ranging from 20,260 to 22,480 J/g. For comparison, the average gross calorific value of coke is estimated to be between 27,000 and 30,000 J/g. Such a significant difference between the heating values of the analyzed biomass and slack results mainly from a large difference in the content of three components, namely carbon, hydrogen, and sulphur.

## 3. Research

### 3.1. Problem Analysis

A typical process in copper metallurgy for recovering contained metals from waste raw materials, including copper slags, is most commonly carried out using electric arc furnaces. This process involves the reduction of oxide compounds of copper and other metals, e.g., lead, using slack or coke as a reducing agent. Assuming that copper and lead in such a material occur mainly in the form of oxides (Cu_2_O and PbO), the reduction reaction is primarily an indirect reduction and can proceed according to the following equation:

MeO + CO = Me + CO_2_ (1)

2MeO + C = 2Me + CO_2_ (2)

Carbon monoxide, which is a reactant in both reactions, is formed in the aggregate as a result of the Boudouard reaction:

CO_2_ + C = 2CO (3)

As previously noted, the components of biomass, including fruit pits, i.e., cellulose, hemicellulose, and lignin, release gases during thermal decomposition that can act as an additional source of reducing agent in the analyzed process. In addition to carbon monoxide, hydrogen and methane can also be considered here [69].

MeO + H_2_ = Me + H_2_O (4)

4MeO + CH_4_ =4Me + CO_2_ +2H_2_O (5)

In this case, the contribution of the direct reduction process using carbon is negligible in the discussed technological process. To determine the probability of these reduction reactions occurring, a thermodynamic analysis of the reduction reactions was performed based on the determined value of the Gibbs free energy change ΔG_0_^T^ in the temperature range of 1250–1400 °C, characteristic of the industrial process, using the equation:

(6) $$∆G_{T}=∆G_{T}^{0}+\mathrm{RTlnU}$$

where R–molar gas constant J/mol K; T—temperature K; and U—equilibrium constant.

Table 2 summarizes the Gibbs free energy results for exemplary reactions of copper and lead oxides that may occur in the slag reduction process.

The above analysis was performed using the HSC thermodynamic database [70]. As can be seen from the data summarized in Table 2, the determined Gibbs free energy for all analyzed reactions took values ranging from −648.4 to −65.8 kJ. The obtained negative ΔG_0_^T^ values indicate that, from a thermodynamic point of view, the occurrence of all these reactions is possible. However, it should be noted that the conducted analysis only demonstrates the probability of the reactions occurring, without providing any information regarding, for example, their kinetics. This is due to the fact that the analyzed reduction reactions can be heterogeneous in nature, and their rate may be determined—in addition to chemical affinity—by many other factors, the most important of which are mass transfer phenomena and the hydrodynamic conditions characterizing the aggregate in which the given process is carried out.

In the works [69,71,72], detailed results are presented for real conditions characterizing the process of copper recovery from flash smelting slag decopperization.

### 3.2. Materials

Biomass in the form of plum, cherry, and apricot pits was used in the study as alternative reducing agents. Their basic chemical composition is presented in Table 3. This biomass was utilized in tests investigating the decopperization process of synthetic slag, the chemical composition of which (in terms of primary elements) is presented in Table 4. The tested slag samples were characterized by a moisture content of 4–6% wt., while the particle size was from 0.5 to 2 mm—measured by the sieve method.

To initially characterize this material, a phase analysis was performed based on the interpretation of a diffractogram obtained using a Seifert-FPM XRD7 X-ray diffractometer [73]. The phase analysis allowed for the identification of the following phases in the slag:

- Diopside CaMgAl_0.5_Si_1.5_O_6_ with a monoclinic structure C2/c (15);
- Leucite K(AlSi_2_O_6_) with a tetragonal structure I41/a (88);
- Akermanite Ca_2_Mg_0.54_Al_0.92_Si_1.54_O_7_ with a tetragonal structure P-421m (113);
- Maghemite Fe_1.966_O_2.963_ with a tetragonal structure P43212 (96);
- Cuprite Cu_2_O with a cubic structure Pn-3m (O_2_) (224);
- Anorthite Al_2_PbSi_2_O_8_ with a monoclinic structure I2/c (15);
- Potassium oxide K_2_O with a tetragonal structure I4/mmm (139);
- Hardystonite Ca_2_Zn(Si_2_O_7_) with a tetragonal structure P-421m (113);
- Massicot PbO with a rhombic dipyramidal structure Pbcm (57).

### 3.3. Research Methodology

**Figure 2:**
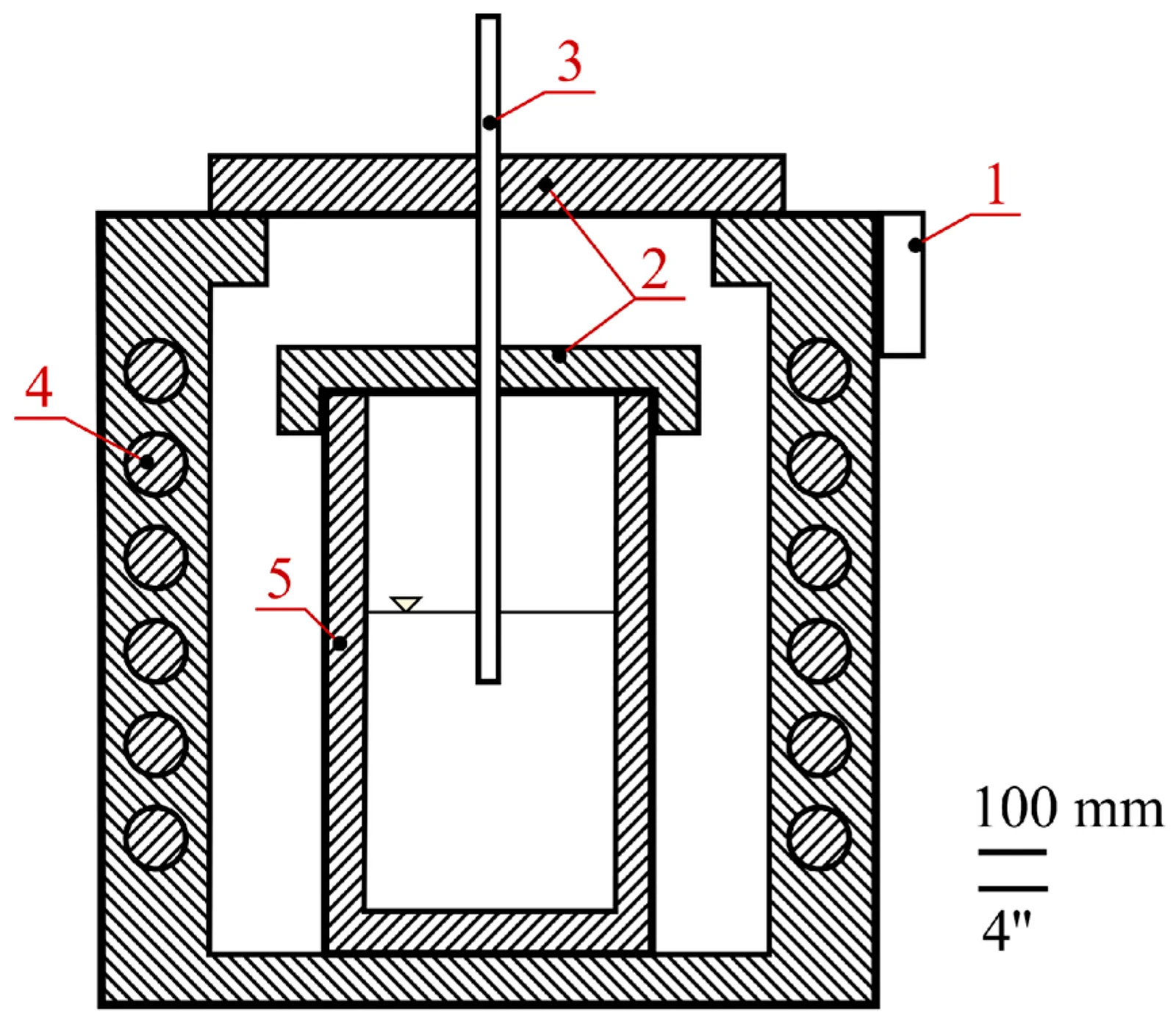
Schematic diagram of the station with an electric resistance furnace for the slag reduction process: 1. Furnace control unit. 2. Ceramic lid. 3. Thermocouple. 4. Resistance heating elements. 5. Crucible with the tested sample [73].

The primary study aimed at determining the suitability of biomass in the process of metal recovery from synthetic slag with a chemical composition similar to the slag from the actual flash-smelting copper production process, involved planning and conducting reduction smelting tests for various variants, in which the effects of the various reducers in the form of biomass, process duration, and temperature were analyzed. The reduction smelting process of the slag with the addition of the biomass-based reducing agent was carried out in a Czylok PT 40/1300 laboratory resistance furnace. A schematic diagram of this device is presented in Figure 2. The use of such a melting aggregate during the tests allowed for easy charging of the feed, as well as the ability to control the process. The reduction process was conducted at a temperature of 1300 °C. This temperature was selected based on the results of the performed slag feasibility tests and data from the copper smelting industry. The mass of the slag sample subjected to testing was 80 g.

The variable parameter in the studies was the process duration, ranging from 1 h to 5 h. The amount of the biomass-based reducing agent added to the process was determined by the similar carbon content of the tested biomasses. In total, the quantity of the added reducing agent was calculated based on the amount of reduced metal oxides in the sample and the corresponding required amount of carbon, the addition of which was calculated in total, based on the stoichiometric reaction (Table 2, add., Bodouard reaction (3)). The data presented in Table 3 show that the elemental carbon content in the analyzed stones/seeds was similar and ranged from 47 to 52 wt. %.

The test procedure involved placing a reducer sample in a crucible with a diameter of 50 mm and a height 80 mm, and then covering it with the slag to be tested. To restrict air access, the crucible was covered with a ceramic lid and placed in the furnace chamber. An opening in the lid allowed for the permanent insertion of a thermocouple to measure the sample temperature. The device heated the sample at a rate of 20 degree Celsius per minute. After reaching the temperature assumed in the experiment, the duration of the process was measured. After the process, the sample was left in the device until it cooled completely. The tests were conducted on three samples in each variant.

The analyses were performed using a Rigaku Primus II X-ray fluorescence spectrometer (WDXRF). Slag samples for XRF measurements were crushed, dried, homogenized, then milled and pressed into pellets with cellulose (30 mm in diameter). For calibration of the XRF, the FluXana standards were used. For quantitative analysis, the Kα line was used for copper and the Lα line for lead. The counting time for peaks was 30 s.

Additionally, these slags were subjected to microscopic analysis using a Hitachi S-3400 N scanning electron microscope (SEM) equipped with an energy-dispersive X-ray spectrometer.

(EDS) Thermo Noran. Structural investigations were carried out using a semiconductor backscattered electron detector (BSE). SEM observations and EDS analyses were performed at a primary electron energy equal to 15 keV and a working distance of 10 mm.

## 4. Results

Studies within one series of time and temperature were conducted for three samples. The results of the experimental tests of copper slag reduction tests, which are the average for individual test series, are presented in Table 5. In addition to the basic input parameters, i.e., mass of copper slag and mass of the reducing agent, it summarizes the products, that is, mass of the (secondary) slag, mass of the obtained alloy, and the copper and lead content. The table also includes the primary parameters adopted in this study, in the form of the degree of decopperization and deleading, in order to compare the efficiency of the given reducing agent.

Five parameters were adopted to evaluate the effectiveness of the analyzed process:

○ Degree of slag decopperization, %;
○ Degree of slag deleading, %;
○ Copper content in the post-process slag, wt. %;
○ Lead content in the post-process slag, wt. %;
○ Amount of smelted alloy, g.

The degree of slag decopperization as well as its deleading was determined from the following relationship:

(7) UCu(Pb)=m0·C0 CuPb−mk·Ck Cu (Pb)m0·C0 Cu (Pb)·100%

where C_0 Cu_ and C_0 Pb_—the initial concentrations of copper and lead, respectively, in the primary slag, wt. %, and C_k Cu_ and Co_k Pb_—the final concentrations of copper and lead in the secondary slag after the reduction process, and wt. %, m_0_ and m_k_—the initial and final slag masses, g.

**Figure 3:**
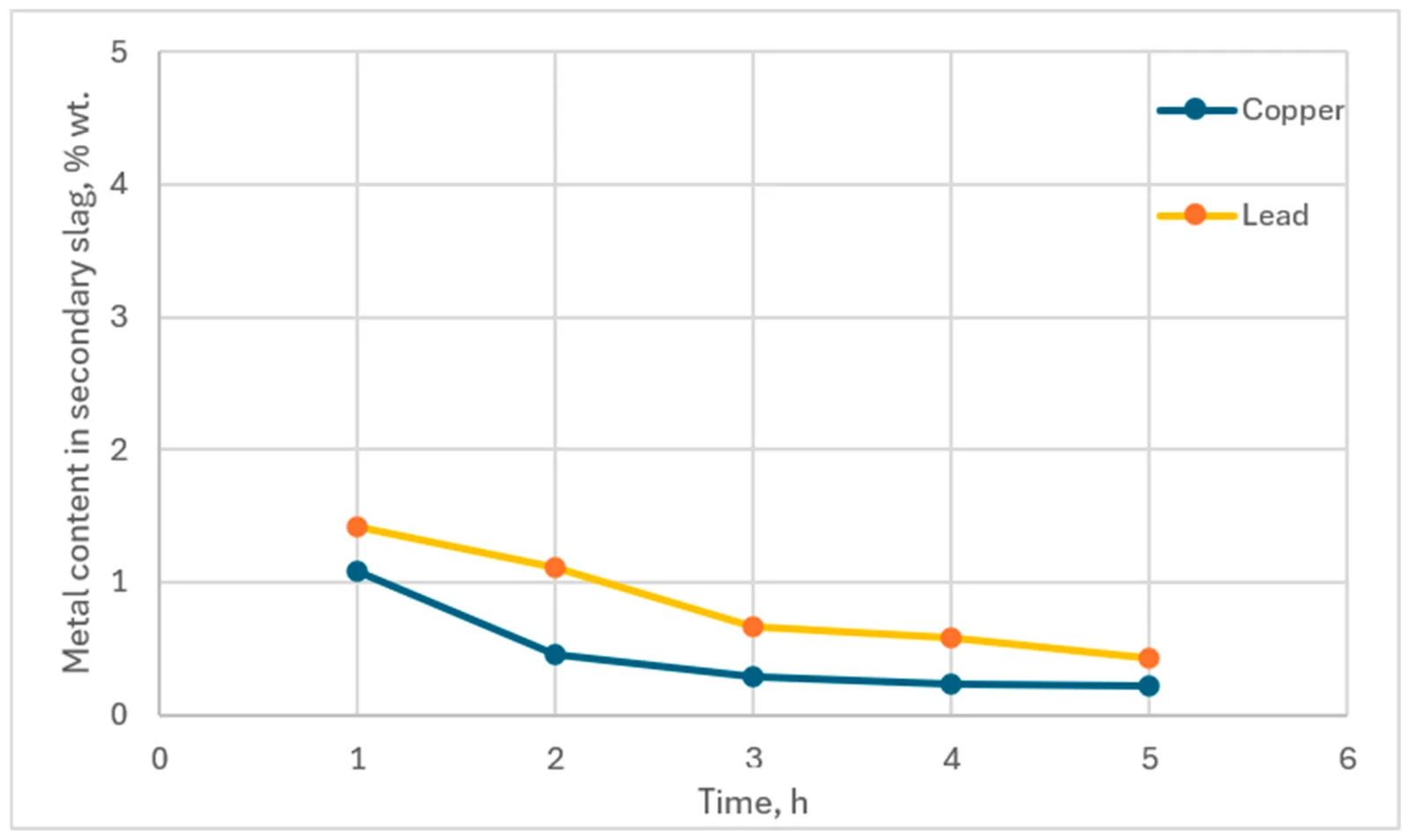
Evolution of Cu and Pb content in the slag reduction process using cherry stones (T = 1300 °C).

**Figure 4:**
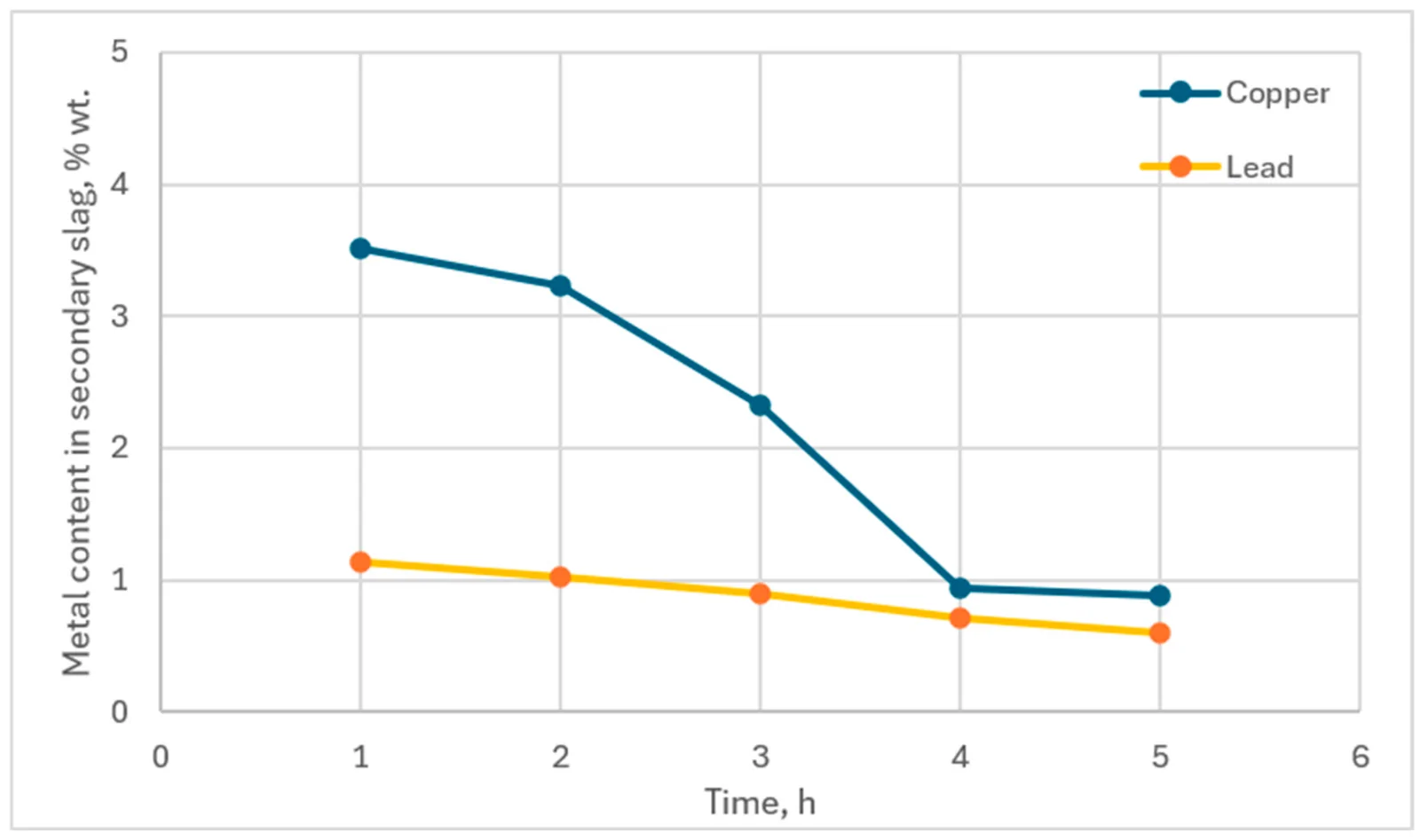
Evolution of Cu and Pb content in the slag reduction process using plum pits (T = 1300 °C).

**Figure 5:**
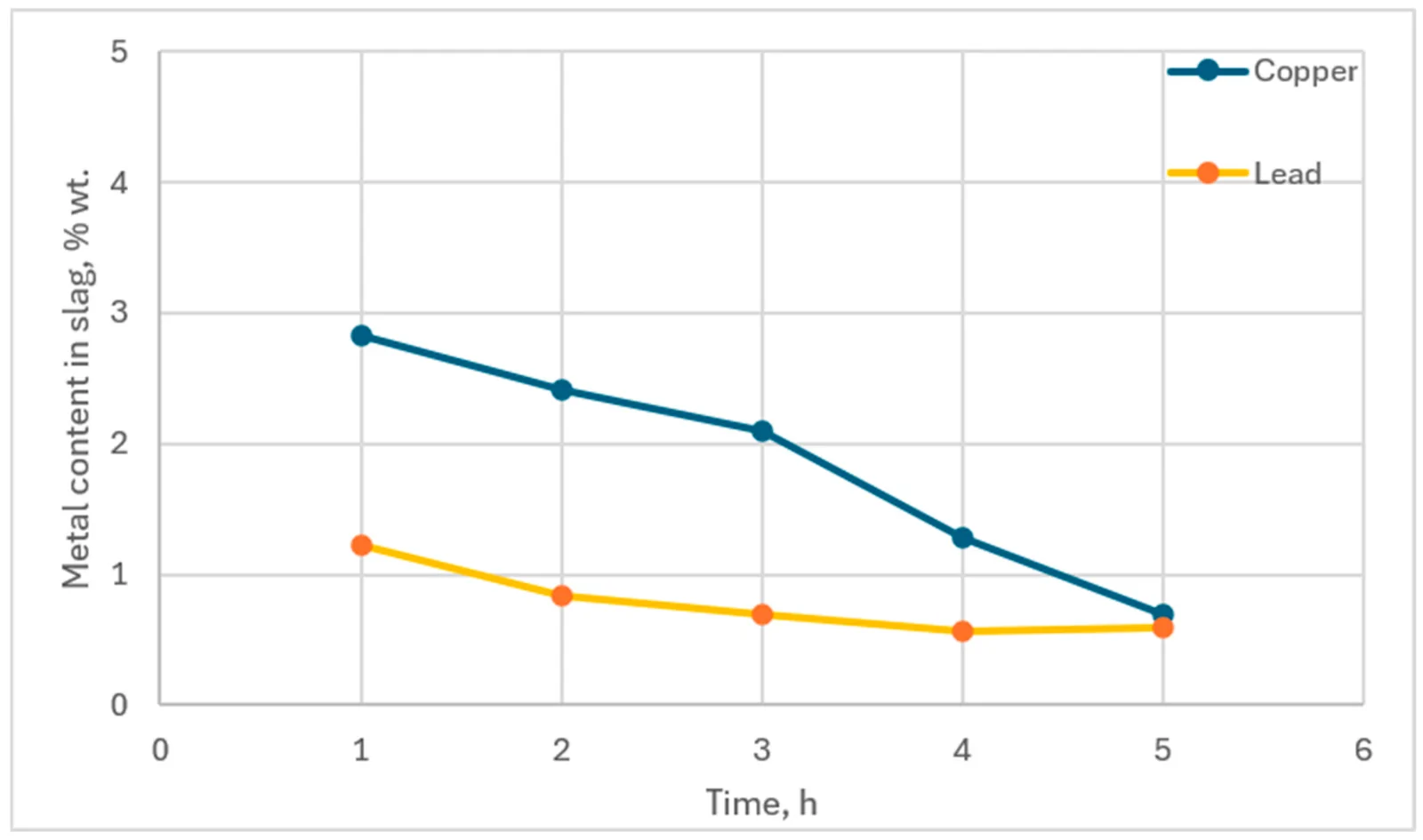
Evolution of Cu and Pb content in the slag reduction process using apricot kernels (T = 1300 °C).

**Figure 6:**
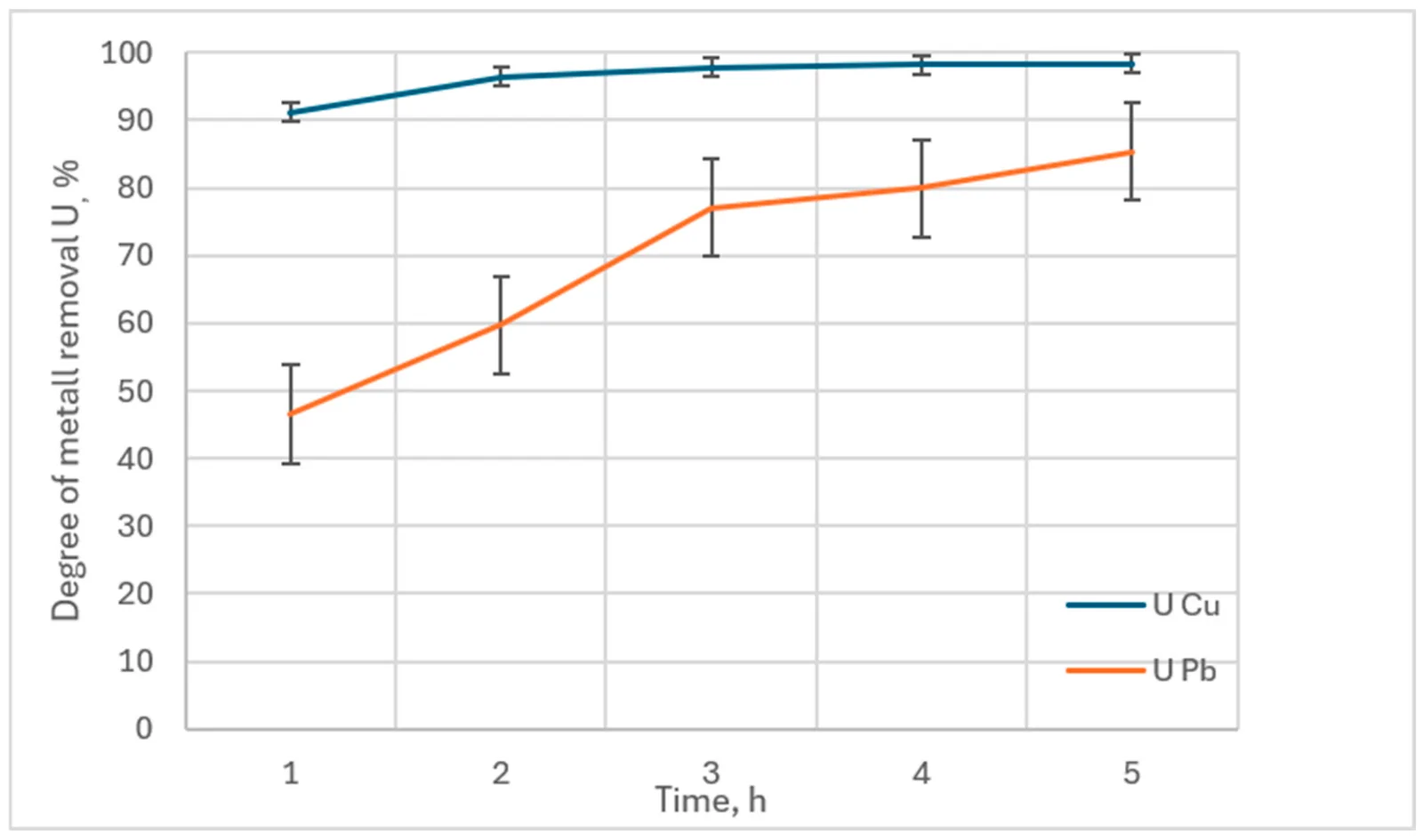
Removal efficiency of Cu and Pb in the slag reduction process using cherry stones (T = 1300 °C).

**Figure 7:**
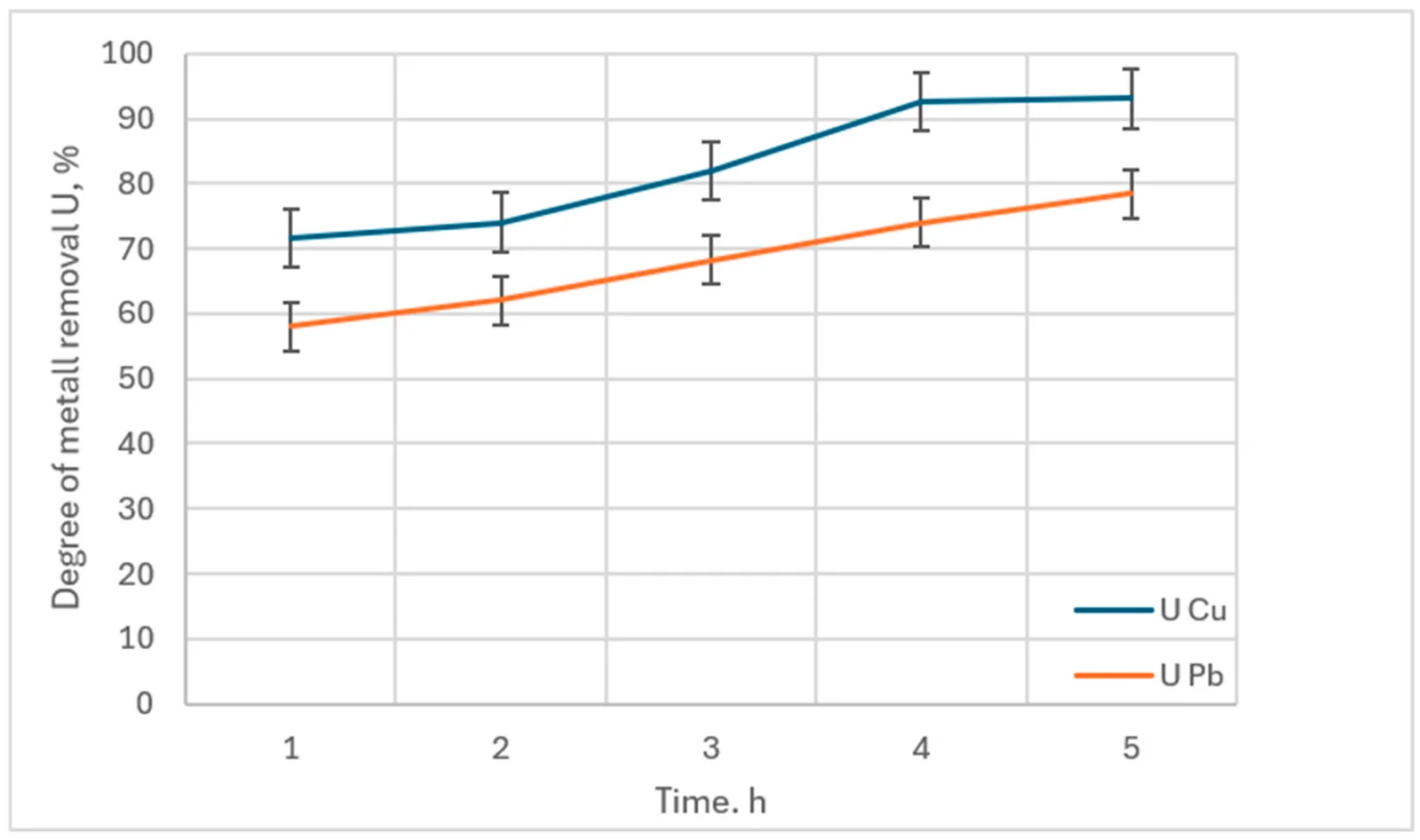
Removal efficiency of Cu and Pb in the slag reduction process using plum pits (T = 1300 °C).

**Figure 8:**
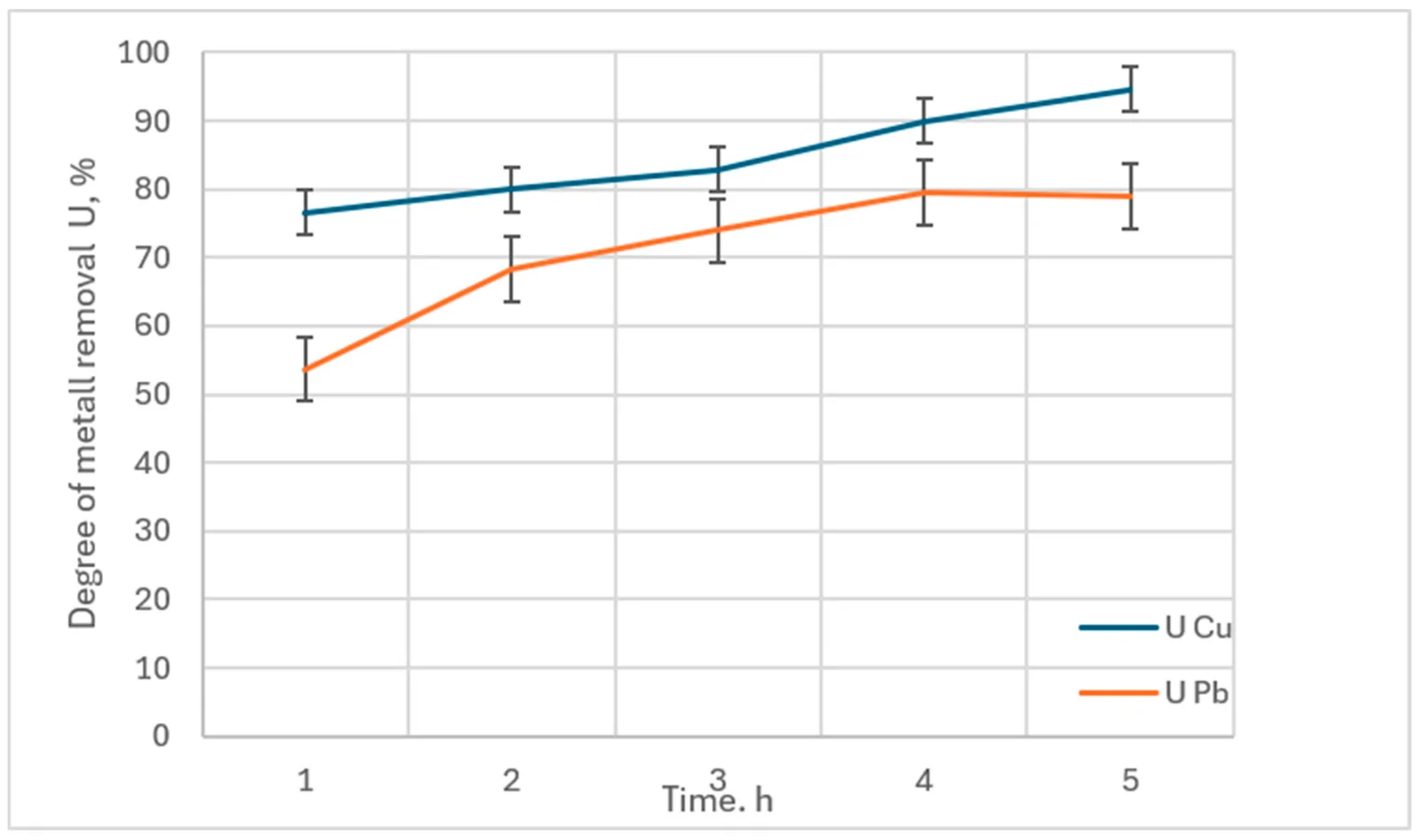
Removal efficiency of Cu and Pb in the slag reduction process using apricot kernels (T = 1300 °C).

An analysis of the copper and lead content in the post-process slag showed a high degree of removal of these metals during the reduction smelting of the primary slag using fruit stone biomass as a reducing agent. For the process conducted using cherry stones, with an initial copper content in the slag of 10.3 wt. %, a reduction in the content of this element in the slag to 1.08 wt. % was achieved for a 1 h process. For the process lasting 5 h, the final copper content in the secondary slag was 0.21 wt. %. This corresponded to an increase in the degree of slag decopperization from 91.1% to 98.4%. This is illustrated by the data presented graphically in Figure 3 and Figure 6. When apricot stones were used as the reducing agent in the process, the lowest copper content achieved in the slag was 0.57 wt. %, which corresponded to a degree of decopperization of 94.6%. Similarly, when plum stones were used in the process, the copper content in the post-process slag reached 0.59 wt. %, and the degree of decopperization was 93.0% (Figure 4, Figure 5, Figure 7 and Figure 8).

When analyzing the obtained results for lead in the slag reduction process, it was found that for all used reducing agents, the degree of removal of this metal fell within the range of 46.6% to 85.4%; however, the best results, similarly to the decopperization process, were achieved when using cherry stones. The post-process slags obtained in all experiments contained lead at a level of 0.43 to 1.42 wt. % (Figure 3, Figure 4 and Figure 5).

**Figure 9:**
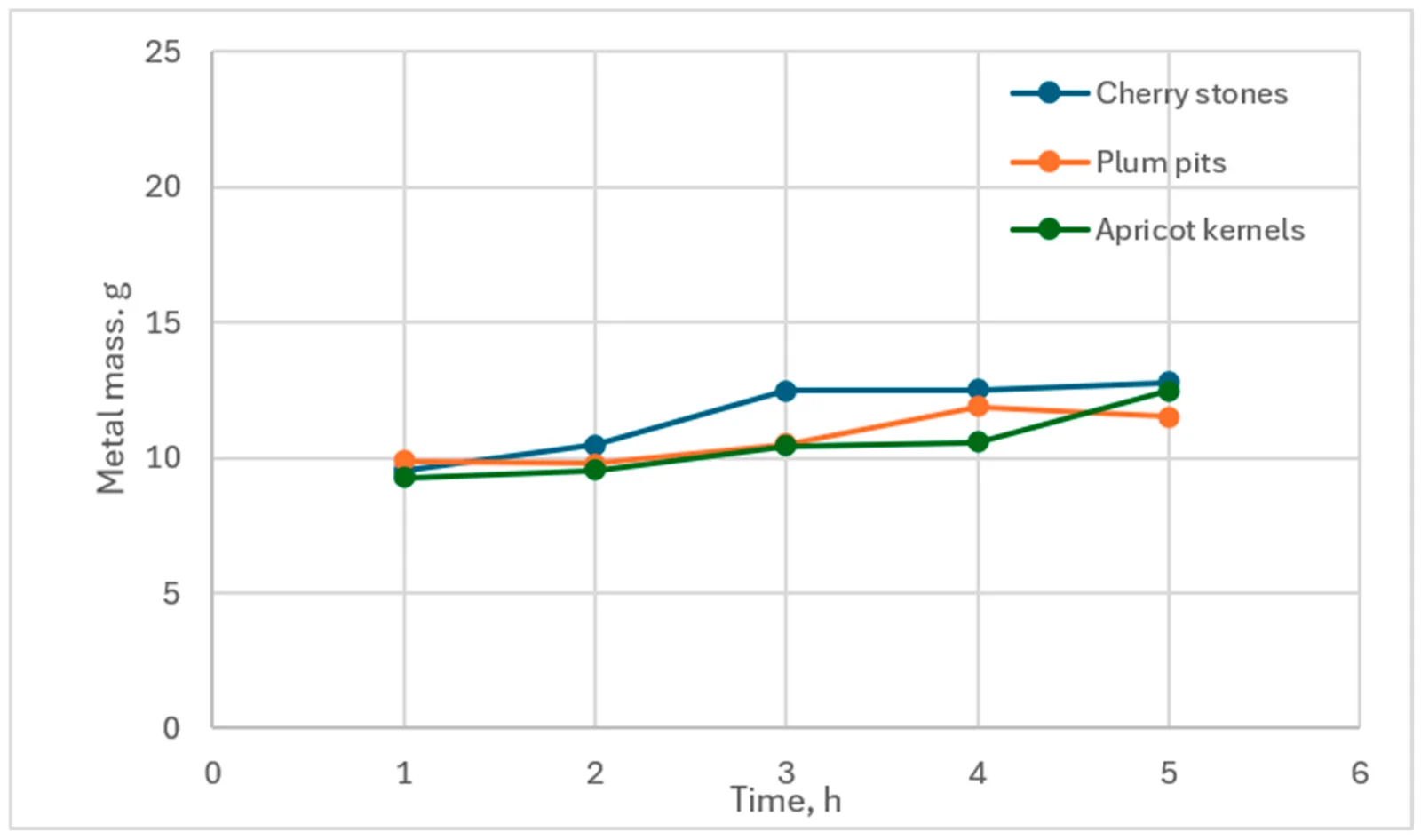
Change in the mass of the reduced alloy depending on the duration of the slag reduction process.

Figure 9 presents the results of the change in the mass of the obtained metal during the reduction process for the used biomass-based reducing agents. The data show that the mass of the alloy formed during the slag reduction process, depending on the smelting duration, ranged from 9.2 g to 12.77 g. No significant differences were found in the masses of the obtained alloy depending on the type of reducing agent used.

**Figure 10:**
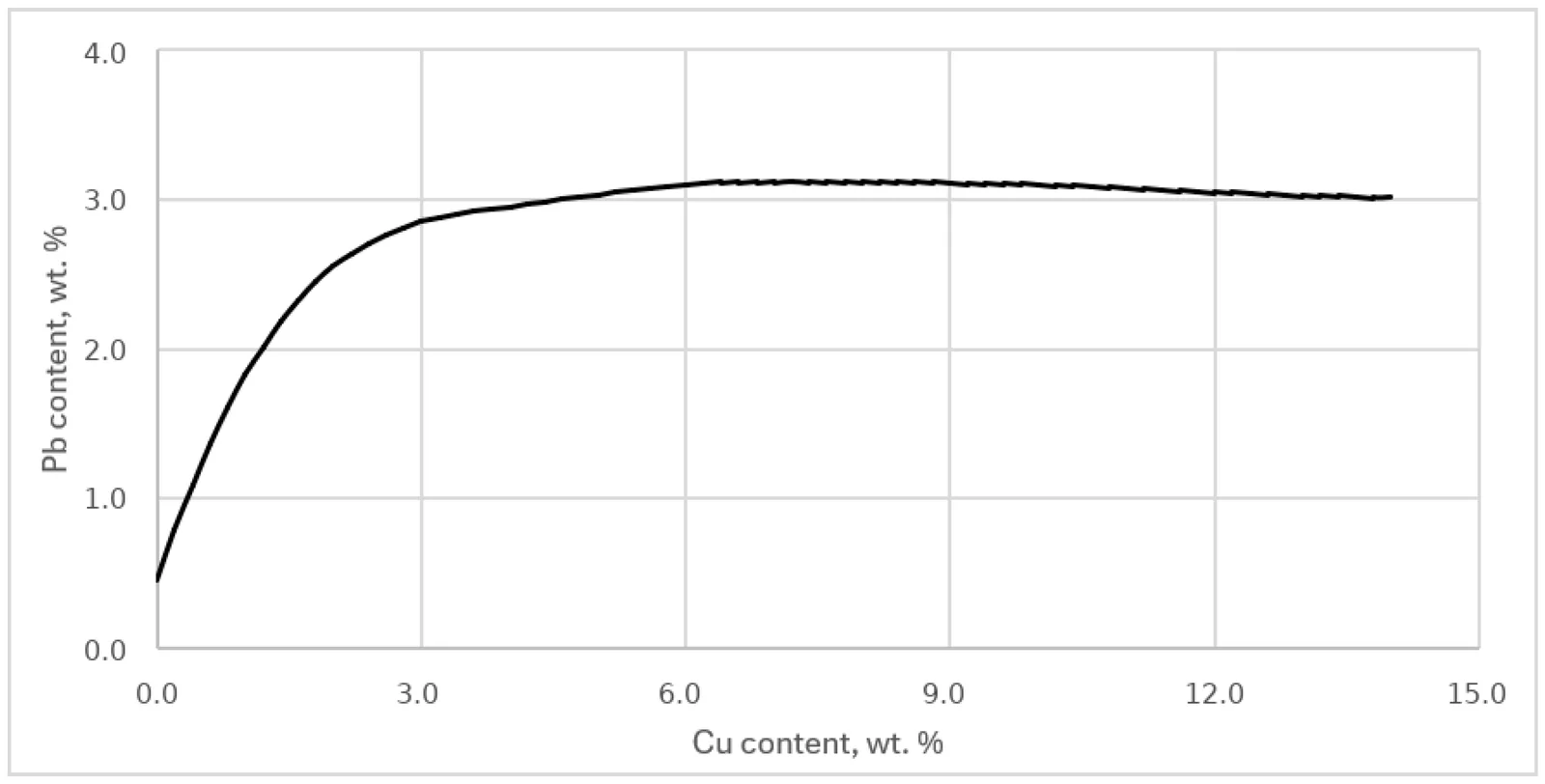
Relationship between the lead and copper content in the slag at equilibrium [76].

Increasing the duration of the reduction process from one to five hours resulted in a roughly 25% increase in the obtained alloy mass, which indicates that the reduction process itself takes place primarily during the initial period of the process. As studies on the reduction of metal oxides contained in copper slags have shown, the first stage of the process involves the reduction of copper oxides, followed by lead oxides, and finally iron oxides; this is consistent with the Gibbs free energy calculations for the individual metal oxides [74,75]. Furthermore, it was demonstrated in [76], based on a thermodynamic analysis, that the reduction of lead oxide contained in the slag begins when the copper content in the form of Cu_2_O oxide drops to <3 wt. %. This is illustrated by the data presented in Figure 10.

**Figure 11:**
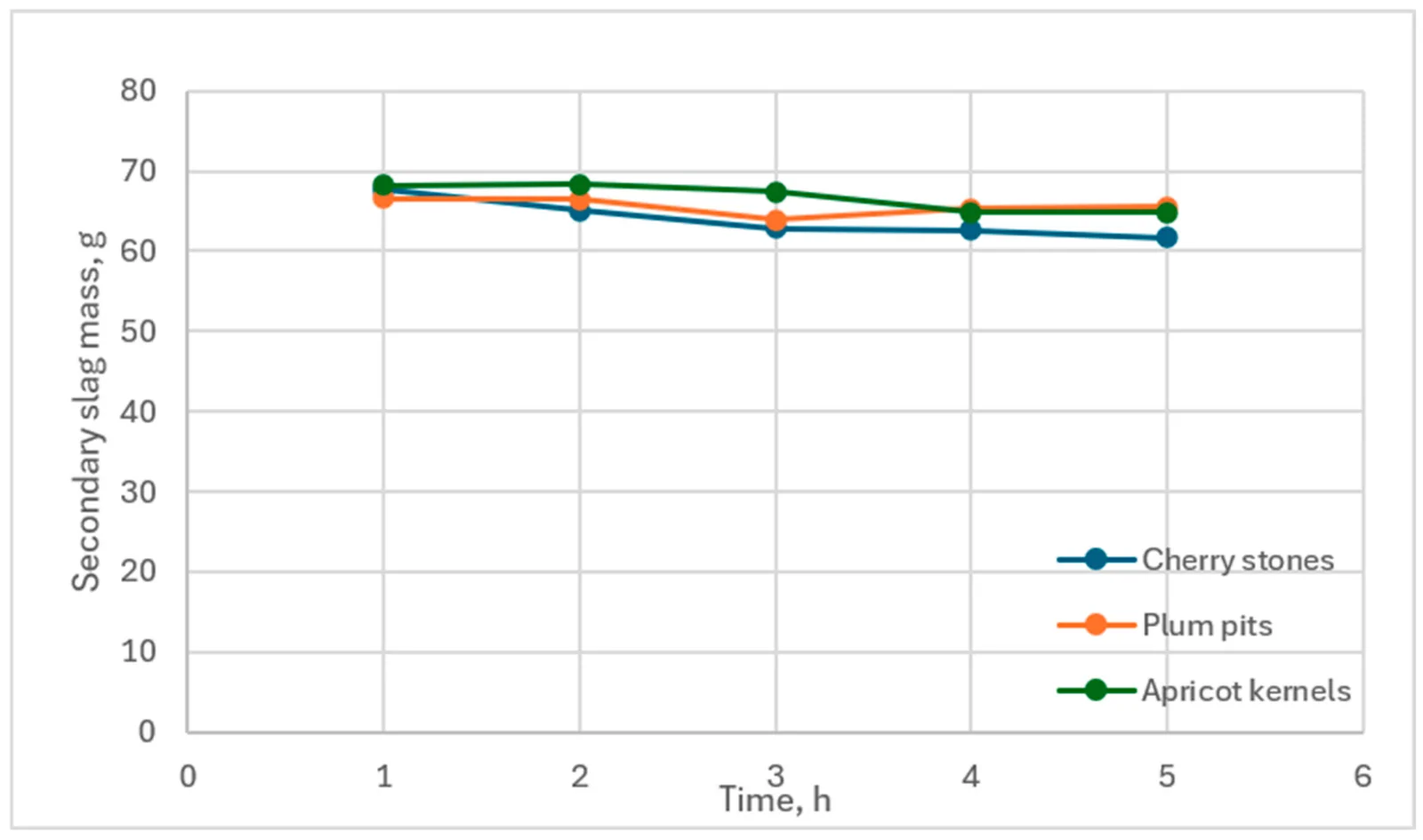
Change in the mass of the post-process slag depending on the duration of the reduction process.

Considering the results regarding the change in copper and lead content, as well as their obtained masses in the initial slag and secondary slag after the process, it was found that after 1 h, the approximate total transfer level of these metals into the alloy ranged from 60% to 80%. For the process carried out over 5 h, this indicator reached 90% to 95%. This confirms that the reduction process of copper and lead oxides essentially takes place during the initial period of the reduction process. It should be noted that in both cases of the process durations used, the higher values of the metal-to-alloy transfer indicator from the slag were associated with reduction using cherry stones. For all experiments, no clear effect of the reduction process duration on the amount of post-process slag formed was observed. For the experiments with the addition of cherry stones as a reductant, the amount of slag formed was lower compared to the trials using the other two bio- reductants (Figure 11). This may be due to the fact that cherry stones proved to be a more effective reductant than plum and apricot kernels.

**Figure 12:**
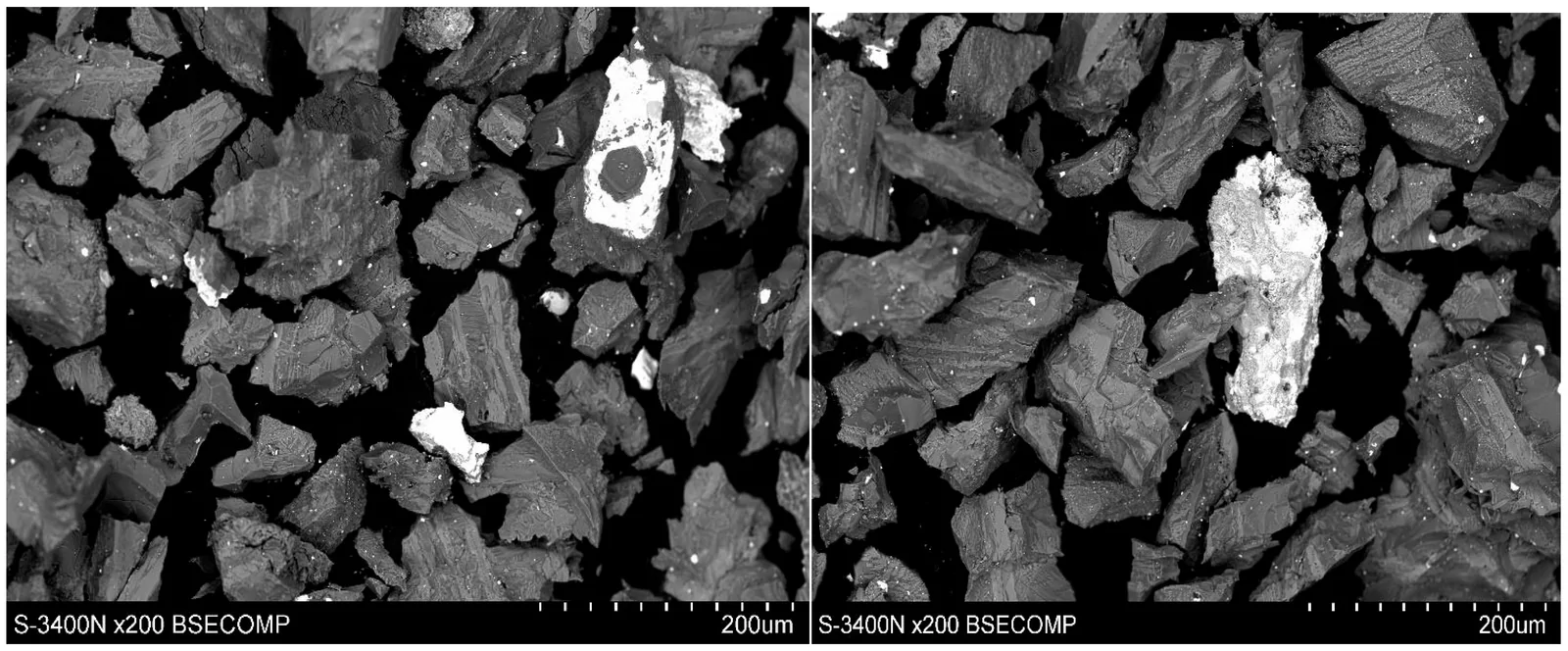
Morphology of the slag after the reduction process.

In order to evaluate the morphology of the obtained slags, a microstructure analysis of the secondary slag was conducted, which revealed the presence of numerous metallic inclusions. Photographs of the microstructure of the obtained slags are presented in Figure 12.

**Figure 13:**
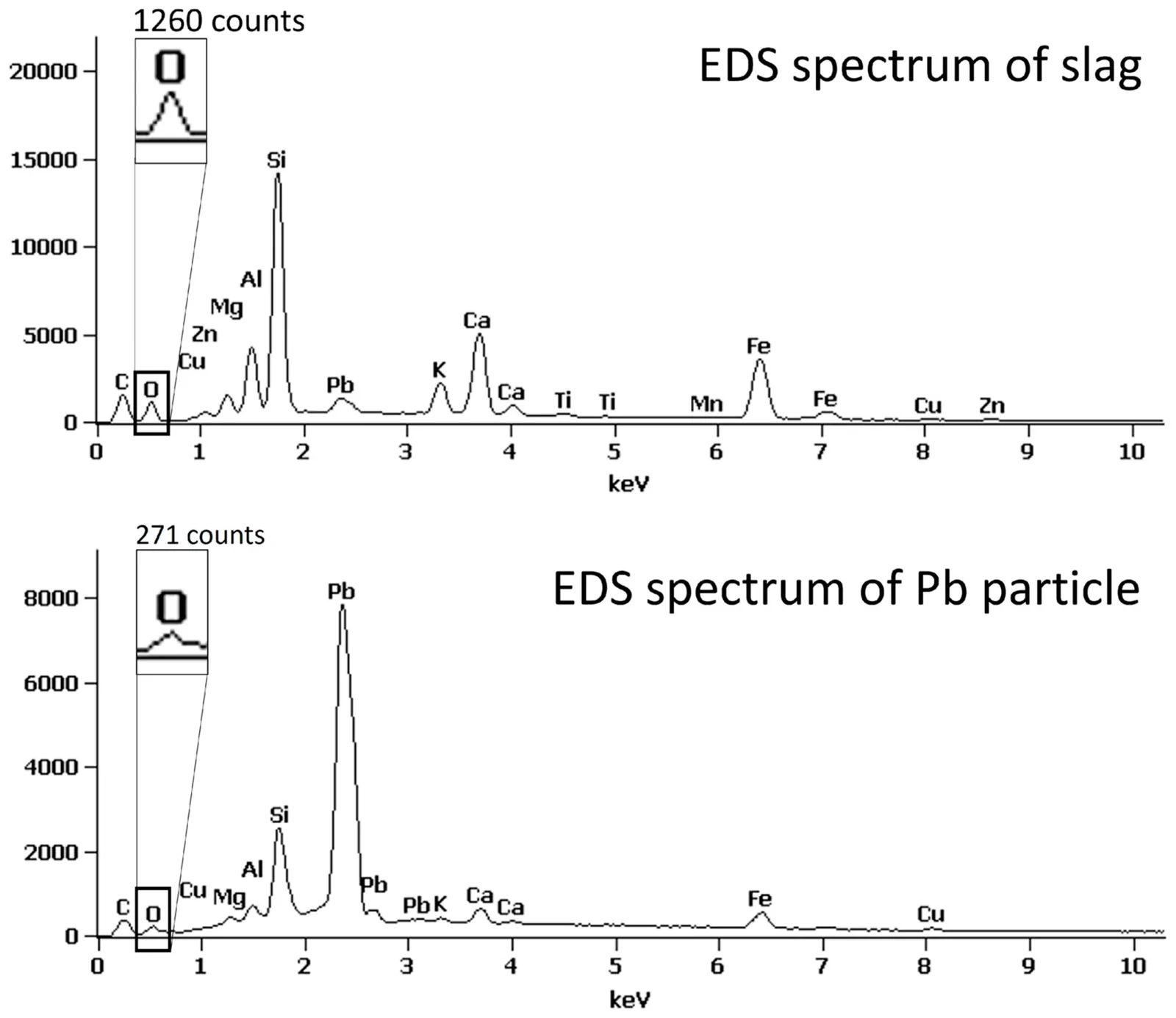
Results of the EDS chemical composition analysis of the secondary slag.

This is confirmed by the results of the EDS chemical composition analysis, which reveal the presence of lead (Pb) in these particles (Figure 13). Furthermore, the EDS spectra show a lower concentration of oxygen in the “white particles” than in the rest of the slag (in the case of the particles, the intensity of the Kα line of oxygen was almost five times lower compared to the slag), suggesting that these particles contain metallic lead, while the rest of the slag is a mixture of oxides.

The observed phenomenon of fine metallic lead inclusions suspended in the slag is unfavourable and significantly reduces the efficiency of the metal reduction and metal alloy smelting process, which is the main product of the analyzed process. The literature data show that in post-reduction secondary slags obtained under industrial conditions, metallic inclusions of both copper and lead can have diameters of up to 100 µm. In this case, their settling velocity in the liquid slag depends mainly on their diameter, as well as the viscosity and density of the liquid slag. It has also been shown that in the case of copper present in the secondary slag, about half of it occurs in oxide form, while the other half is in the form of suspended fine micro-droplets of metallic copper [74,75,76,77,78,79]. Increasing the efficiency of metallic droplet sedimentation from the slag phase can be achieved by increasing the volume of the droplets through their coalescence.

This can be achieved, for example, by increasing the collision frequency of the suspended metal droplets. Under industrial conditions, such an effect is achieved by inducing turbulent motion of the liquid slag, for example, through the addition of calcium carbonate. Its thermal decomposition generates a gas, the flow of which intensifies the mixing process.

## 5. Summary

Based on the positive results of laboratory tests, it is advisable to conduct preliminary larger-scale tests enabling their industrial application, to determine the appropriate parameters for the large-scale process, such as the amount of coke that can be replaced by seed-stone biomass.

At this stage of the research, the reference point allows us to assume that the biomass material used can be an alternative to coke if the reduction process allows for reducing the copper content in the industrial slag below 0.6 wt. % Cu by mass. Although from a thermodynamic point of view it is possible to further reduce the concentration of the main elements, economic considerations make further process unprofitable.

The experimental research results presented in the article, as well as their analysis, have shown that fruit seed-stones can be used in the metallurgical industry as an alternative reducing agent in the process of recovering copper and lead from copper slags. This is mainly determined by their relatively high carbon content and the fact that the main components of biomass, i.e., cellulose, hemicellulose, and lignin, additionally release reducing gases such as hydrogen, methane, and carbon monoxide during thermal decomposition.

The determined Gibbs free enthalpy indicates that the indicated chemical reactions of reduction of the base metals Cu and Pb obtain values reaching the level of −600 kJ/mol for methane reduction. The calculated index of the degree of S_Cu_ copper removal varies in the range from approx. 71% in the process lasting 1 h with the addition of a reducer in the form of plum seeds to a level above 98% in a process lasting 5 h with the addition of a reducer in the form of cherry seeds. Attention should be paid here to the specific form of copper in slag, where after the reduction process the metal occurs in the form of suspended droplets of microscopic sizes, which hinder the process of sedimentation and separation from the slag part. From the point of view of hydrodynamics, this type of system requires the introduction of a liquid into motion that intensifies the mechanism of coagulation and combination of metal droplets into larger ones to activate the sedimentation process [69,71,72]. In the biomass reducers used in the studies, the difference in the efficiency results obtained may be caused by the released hydrocarbons, which, in addition to having the role of the reducer, move deep into the charge, activating the reduction processes. In the case of lead, the value of the degree of S_Pb_ is contained from approx. 48% in the reduction process for 1 h and increases to over 85% after 5 h of the process with the addition of a reducer in the form of cherry seeds.

The obtained removal efficiencies may be subject to an error of 5–10% due to the accuracy of X-ray fluorescence (XRF) analysis and the sampling procedure. After the process, the material was ground to a particle size of 0.1–0.5 mm, and a 5 g sample was collected for analysis using the quartering method.

The conducted research demonstrated the possibility of decopperization of the slag from a level of 10.3 wt.% to 0.21 wt.%. In the case of lead, the removal rate of this metal from the slag ranged from 2.25 to 0.48 wt. %. The results gained become particularly significant if the copper content in the process slags is related to the copper content in the ore, where the percentage of this element hovers around 1 wt. % [80].

Additionally, it reduces environmental emissions of pollutants such as SO_2_ and NO_x_, while on the other hand, it can intensify the reduction process of metal compounds itself. The latter results from the fact that the main components of biomass, i.e., cellulose, hemicellulose, and lignin, additionally release reducing gases such as hydrogen, methane, and carbon monoxide during thermal decomposition.

Based on data (ETS2—European Commission), the current cost level for the emission of 1 ton of CO_2_ is approx. 90 €, while in 2030, the emission fee for 1 ton of CO_2_ is expected to reach 120 €–180 € [81].

Taking the above into account, given the estimated coke consumption in the copper production process, it is assumed that the annual consumption of coke in this production amounts to 20,000 Mg. Assuming that 90% of the carbon is converted into CO_2_, the emission will amount to approx. 48,000 Mg of CO_2_, which results in a total cost of approx. 4.3–8.6 million euros/year. Considering the current trends, this amount could double within the next few years.

For industrial applications, in order to meet the requirements of the technological process, this analysis should be significantly expanded to include the costs associated with the construction of a seed-stone agglomeration plant capable of producing material in the required quantity and quality, as well as the costs and methods associated with raw material storage.

## Figures and Tables

**Table 1:** Directions for the utilization of biomass from fruit pits.

Type of Biomass	Method of Utilization	Literature
**Cherry stones**	Direct use as fuel	[3]
	Substrate for the production of liquid and gaseous fuels	[4,5]
	Activated charcoal production	[6,7]
	Component of animal feed	[7]
	Cosmetics production	[8]
**Plum pits**	Biofuel production	[9,10]
	Pellet manufacturing	[11]
	Cooking oil production	[12]
	Biochar production	[13,14]
	Purification of aqueous solutions	[14,15,16,17]
	Composite panel manufacturing	[18]
**Apricot kernels**	Biofuel production	[14,19,20,21,22,23]
	Component in dietary supplements and medication	[24,25,26,27]
	Activated charcoal production	[28,29]
	Cosmetics production	[30]
	Pharmaceutical industry	[30,31]

**Table 2:** Estimated ΔG_0_^T^ values of Cu_2_O and PbO reduction reactions in the temperature range of 1250–1400 °C [70].

No	Reaction	ΔG_0_^T^, kJ			
		1250 °C	1300 °C	1350 °C	1400 °C
1	2Cu_2_O + C = 4Cu(l) + CO_2_ (g)	−309.429	−313.721	−317.969	−322.172
2	Cu_2_O + CO(g) = 2Cu(l) + CO_2_ (g)	−83.945	−81.878	−79.796	−77.701
3	Cu_2_O + H_2_(g) = 2Cu(l) + H_2_O (g)	−104.236	−103.508	−102.756	−101.982
4	4Cu_2_O + CH_4_(g) = 8Cu(l) + CO_2_(g) + 2H_2_O (g)	−623.957	−632.218	−640.377	−648.439
5	2PbO + C = 2Pb(l) + CO_2_(g)	−278.593	−285.252	−291.860	−298.421
6	PbO + CO(g) = Pb(l) + CO_2_(g)	−68.527	−67.643	−66.742	−65.825
7	PbO + H_2_(g) = Pb(l) + H_2_O(g)	−88.818	−89.273	−89.702	−90.107
8	4PbO + CH_4_(g) = 4Pb(l) + CO_2_(g) + 2H_2_O (g)	−562.287	−575.278	−588.161	−600.937

**Table 3:** Main component content of the biomass utilized in the research [55,56,57,58,59,60,61,62,63,64].

Type of Biomass	Component, wt. %			
	C	S	H	O_2_
Apricot kernels	51.62	0.01	7.43	41.1
Cherry stones	47.41	0.06	7.30	37.62
Plum pits	47.75	0.02	7.15	43.02
Coke	85–95	0.5–2	0.5–1	0.2–1.5

**Table 4:** Chemical composition of the synthetic copper slag.

Slag component	Cu	Pb	Fe	SiO_2_	CaO
Component content, wt. %	10.3	2.25	11.1	34.5	14.1

**Table 5:** Average research results of copper slag reduction tests with fruit pits.

No	Initial Slag Mass	Reducing Agent Type	Reducing Agent Mass	Time	Metal Mass	Secondary Slag Mass	Secondary Slag Content		Metal Removal Rate	
							Cu	Pb	U _Cu_	U _Pb_
	g		g	h	g	g	wt. %		%	
1	80	Cherry stones	18.5	1	9.50	67.67	1.08	1.42	91.1	46.6
2				2	10.84	65.04	0.45	1.11	96.4	59.8
3				3	12.46	62.81	0.29	0.66	97.8	77.1
4				4	12.55	62.60	0.23	0.58	98.3	79.9
5				5	12.79	61.65	0.21	0.43	98.4	85.4
6	80	Plum pits	18.5	1	9.79	66.60	3.52	1.13	71.6	58.1
7				2	9.77	66.43	3.22	1.03	74.0	62.1
8				3	10.53	63.88	2.32	0.89	82.0	68.3
9				4	11.91	65.26	0.94	0.72	92.6	74.0
10				5	11.53	65.52	0.88	0.59	93.0	78.4
11	80	Apricot kernels	18.5	1	9.30	68.19	2.82	1.22	76.6	53.7
12				2	9.58	68.28	2.41	0.83	80.0	68.4
13				3	10.46	67.41	2.09	0.70	82.9	73.9
14				4	10.56	64.79	1.27	0.57	90.0	79.5
15				5	12.40	64.77	0.69	0.59	94.6	78.9

## Data Availability

https://omega.polsl.pl/index.seam, accessed on 1 July 2026.
